# 药学综合创新实验：分子印迹聚合物制备及其在体内药物分析样品前处理中的应用

**DOI:** 10.3724/SP.J.1123.2025.08009

**Published:** 2026-05-08

**Authors:** Yuanyuan JIANG, Ruiling GUO, Zhihua SONG, Zongliang LIU, Yutong FAN, Hui XU

**Affiliations:** 1.烟台大学药学院，山东 烟台 264005; 1. School of Pharmacy，Yantai University，Yantai 264005，China; 2.烟台理工学院食品与生物工程学院，山东 烟台 264003; 2. School of Food and Bioengineering，Yantai Institute of Technology，Yantai 264003，China

**Keywords:** 药学综合创新实验, 分子印迹聚合物, 生物样品前处理, 结构表征, 效能评价, comprehensive pharmaceutical innovation experiment, molecularly imprinted polymers （MIPs）, biological sample pretreatment, structural characterization, efficiency evaluation

## Abstract

在全面推进新药科建设和大学生创新创业训练的背景下，我们针对药物分析领域复杂生物样品分析的前处理技术难题，设计了一项将药物分析、高分子化学、分析化学等药学相关学科方向的理论与技术有机融合的药学综合创新实验。实验选择以先进的分子印迹技术为工具，以重要的内源性代谢物胆酸为研究对象，采用沉淀聚合法制备胆酸分子印迹聚合物，应用扫描电镜和红外光谱分析进行结构表征，阐明印迹位点和形成机制，并利用高效液相色谱法和液相色谱-质谱联用分析方法，系统评价材料的吸附性能、选择性及对血浆等生物样品中微量胆酸的分离富集效能。该实验涵盖了从材料设计到合成制备、结构表征、应用性能评价等丰富内容，体现了高阶性、创新性和挑战度。通过专业实践与学科前沿和课程思政的有机结合，切实提升药学专业学生的创新思维和综合实践能力，激发学生的社会和职业责任意识，促进培养高素质应用型药学人才，是全人教育的有益实践。

## Abstract (en)

Under new pharmaceutical science initiatives and innovation training， we address complex biological sample analysis. A comprehensive pharmaceutical experiment was designed for this purpose. It integrates pharmaceutical analysis， polymer chemistry， and analytical chemistry. Advanced molecular imprinting technology （MIT） serves as the key tool. Cholic acid， a significant endogenous metabolite， is selected as the template. Cholic acid molecularly imprinted polymers （CA-MIPs） are synthesized via precipitation polymerization. Their structure is characterized using scanning electron microscopy （SEM） and Fourier transform infrared spectroscopy （FT-IR）. The imprinting site and formation mechanism is elucidated. Adsorption performance is evaluated by high performance liquid chromatography （HPLC） and liquid chromatography-mass spectrometry （LC-MS）. Selectivity and enrichment efficiency in plasma are assessed. The experiment covers material design， synthesis， and characterization. It also includes application performance evaluation. This demonstrates high-level innovation and challenge. The practice enhances students’ innovative thinking and practical skills. It fosters social and professional responsibility. The project cultivates high-quality applied pharmaceutical talents. This represents a beneficial practice in holistic education.

高质量发展是全面建设社会主义现代化国家的首要任务，高等药学教育对于生命健康领域的高质量发展和打造新质生产力至关重要^［1，2］^。作为学科融合创新科研训练体系的重要环节，各高校在药学专业人才培养方案的专业核心实践课程平台中普遍开设“药学综合创新实验”，使学生及时了解生物医药行业相关领域的前沿新理论、新材料、新技术、新方法，在药物研发、生产、应用的真实场景中培养学生的实践能力、创新思维和综合素质^［3］^。

分子印迹技术（molecular imprinting technology， MIT）是一种为获得在空间结构和结合位点上与目标分子高度匹配的聚合物的新兴实验制备技术，基于MIT制备的分子印迹聚合物（molecularly imprinted polymers， MIPs）尤其适用于基质复杂的体内药物分析样品前处理，受到广泛关注^［4-6］^。与此同时，MIPs的设计、制备及其表征、应用融合了高分子材料学、物理化学、分析化学等药学相关学科领域的基础理论和实验技能，是兼具高阶性、创新性和挑战度的综合创新实验教学资源。有鉴于此，我们结合教学团队前期针对MIT取得的科研成果^［7-9］^，以解决在药物代谢组学研究中普遍存在样品基质效应强、低含量目标分析物定性定量分析的困难^［10，11］^为目标，设计了一项药学综合创新实验。该实验以常见内源性代谢物胆酸为模板，研究内容涵盖分子印迹聚合物制备、表征及在血浆等体内药物分析样品前处理中的应用效能评价。采用沉淀聚合法成功合成了胆酸分子印迹聚合物（CA-MIPs），并利用扫描电子显微镜（SEM）和红外光谱（FT-IR）对其形貌与结构进行表征，初步阐明了印迹位点的形成机制。为系统评价材料性能，研究结合高效液相色谱（HPLC）与液相色谱-质谱联用技术（LC-MS）开展分析：LC-MS融合色谱分离与质谱检测优势，准确测定吸附过程中胆酸（CA）的浓度变化，用于评估材料的吸附性能；HPLC凭借高分离效率与灵敏度，实现对血浆样本中CA的高选择性富集与准确定量检测，从而保障了选择性评价的可靠性。实验设计充分体现了跨学科综合的特点，强调了药学实践与行业前沿和课程思政的有机结合，从而促进产教融合的创新药学人才培养，切实服务科教兴国战略。

## 1 实验部分

### 1.1 仪器、试剂与材料

JSM-7900F扫描电子显微镜（日本电子株式会社），JEM-1400plus透射电子显微镜（TEM，日本电子株式会社），FT-IR360傅里叶变换红外光谱仪（日本岛津公司），AB SCIEX Triple Quad 4500Q液相色谱-质谱联用仪（美国SCIEX公司），Thermo Scientific高效液相色谱仪（配置紫外可见检测器，美国赛默飞世尔科技公司），TGL-20W台式高速冷冻离心机（湖南湘仪实验室仪器开发有限公司），IKAKS4000i控制型振荡摇床（德国IKA公司）。

甲基丙烯酸（MAA，分析纯）、乙二醇二甲基丙烯酸酯（EGDMA，分析纯）、甘草次酸（GA，≥98%）、人参皂苷Rg1（≥98%）均购自上海阿拉丁生化科技股份有限公司，乙腈（ACN，色谱纯）、甲醇（MeOH，色谱纯）购自德国默克生物科技有限公司，胆酸（≥98%）、鹅去氧胆酸（CDCA，≥98%）、地塞米松（DXMS， ≥99%）、秋水仙碱（COL，≥99%）购自上海安耐吉医药化学有限公司，偶氮二异丁腈（AIBN，分析纯）、乙酸（AcOH，分析纯）、甲酸（FA，色谱纯）、丙酮（ACE，分析纯）购自国药集团化学试剂有限公司、超纯水（UPW）购自杭州娃哈哈集团有限公司。

### 1.2 分析检测项目与仪器条件

红外光谱分析^［12］^：采用溴化钾压片法制备固体样品，仪器分辨率4.0 cm^-1^，扫描次数32，光谱扫描的波数范围4 000~500 cm^-1^。

电镜观察：取5 mg左右MIPs样品，用水分散后吸取10 μL至铜网中，晾干，滴加磷酸钨染液染色，晾干，置于TEM中观察和图像采集；取1 mg左右的MIPs样品，用电胶布置于样品台上，喷金，置于SEM中观察和图像采集。

HPLC分析：采用Agilent TC-C18色谱柱（150 mm×4.6 mm，5 μm），柱温30 ℃，进样量10 μL，流速1.0 mL/min，各分析物的流动相和检测波长见表1。

**表1:** 各分析物HPLC分析的流动相与检测波长^［13-16］^

Analyte	Mobile phase （volume ratio）	Detection wavelength/nm	Ref.
Cholic acid （CA）	ACN-UPW （60∶40）	192	［^13^］
Chenodeoxycholic acid （CDCA）	ACN-UPW （80∶20）	210	［^13^］
Glycyrrhetinic acid （GA）	ACN-0.1% formic acid in water （80∶20）	254	［^15^］
Colchicine （COL）	MOH-0.1% formic acid in water （60∶40）	350	［^14^］
Dexamethasone （DXMS）	ACN-UPW （49∶51）	240	［^16^］

UPW： ultrapure water.

LC-MS分析^［17-19］^：色谱分离采用Waters Acquity UPLC BEH C18色谱柱（100 mm×2.1 mm，1.7 μm），柱温40 ℃，进样量5 μL，进样温度4 ℃，流动相为0.1%甲酸水（A）-甲醇（B）混合溶液，流速0.4 mL/min，梯度洗脱程序如下：0~4.5 min，30%A~25%A；4.5~5 min，25%A~30%A。质谱检测采用电喷雾离子源，负离子模式，喷雾电压4 200 V，碰撞气0.028 MPa，气帘气0.21 MPa，雾化气、辅助气0.34 MPa，离子源温度500 ℃，一级全扫描/数据依赖的二级扫描（Full MS/dd-MS^2^）模式扫描，CA *m/z* 407.40、CDCA *m/z* 391.41。

### 1.3 MIPs制备

分子印迹聚合物的制备方法有很多，其中经典的沉淀聚合法能够通过调控溶剂极性和交联剂比例直接生成粒径均一的微球，实现MIPs形貌和孔结构的精确设计，同时避免后续工艺对印迹位点的破坏^［20，21］^。因此，本实验以MAA为功能单体、EGDMA为交联剂，采用沉淀聚合法制备了CA-MIPs，以保证MIPs的传质效率和吸附容量^［21，22］^。

如图1所示，MIPs制备过程简述如下。取模板分子CA（0.01 mmol）溶于丙酮（20 mL），按一定比例加入功能单体MAA（0.04 mmol），将上述混合溶液于室温条件下预组装12 h；依次加入交联剂EGDMA（2 mmol）、引发剂AIBN（10 mg），超声10 min，氮气保护下65 ℃油浴反应24 h，旋蒸除溶剂，残渣加甲醇（含10 %乙酸）30 mL，室温下振荡洗脱30 min（300 r/min），离心10 min（8 000 r/min），弃去上清液，反复6次；残渣加乙腈分散，离心5 min（8 000 r/min），弃去上清液，反复3次，所得沉淀在60 ℃下干燥24 h，即得MIPs。作为对照，除不添加CA外，按上述完全相同步骤制备空白印迹聚合物（NIPs）。

**图1:**
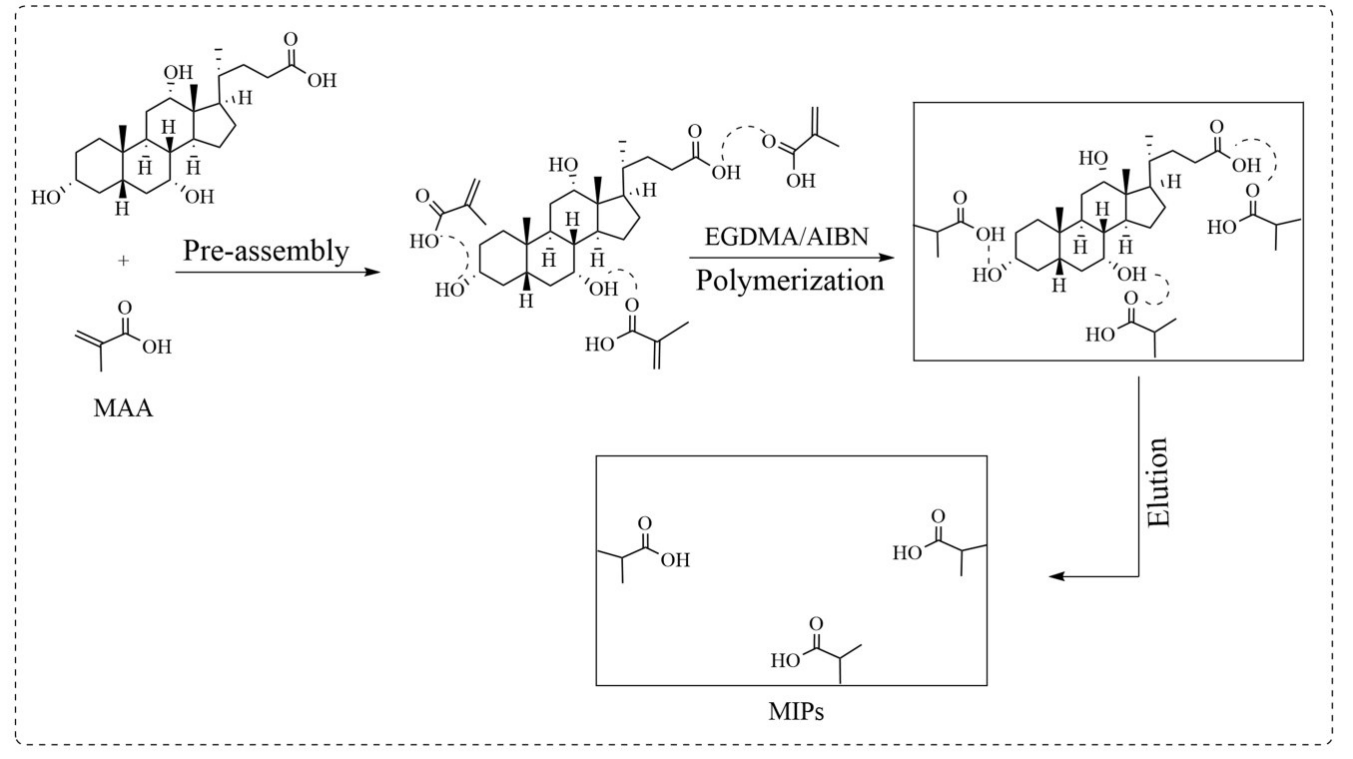
MIPs的合成示意图

### 1.4 吸附性能评价

等温吸附特性：称取25 mg CA，置于25 mL棕色量瓶中，加甲醇溶解，制成1 mg/mL的储备溶液；继续以丙酮为溶剂稀释，得到质量浓度0.5~100 ng/mL的CA系列标准溶液。在西林瓶中加入5 mg聚合物和2 mL CA标准溶液，室温下摇床振荡2 h；离心，吸取上清液，氮吹至干，残渣加流动相0.1%甲酸水-甲醇（30∶70，体积比）复溶，LC-MS法测定CA含量，计算MIPs对CA的结合量，按式（1）计算吸附容量，按式（2）、（3）拟合Langmuir和Freundlich模型方程：


$$Q_{e}=\frac{（C_{0}-C_{e}）V}{m}$$ (1)

$$Q_{e}=\frac{Q_{m}K_{L}C_{e}}{1+K_{L}C_{e}}$$ (2)

$$Q_{e}=K_{F}{C_{e}}^{1/n}$$ (3)

其中，*Q*_e_为吸附容量（mg/g），*C*_0_为CA的初始质量浓度（ng/mL），*C*_e_为吸附平衡时CA的质量浓度（ng/mL），*V*为CA溶液加入体积（mL），*m*为MIPs加入量（mg），*Q*_m_为聚合物最大吸附量（mg/g），*K*_L_为Langmuir吸附常数，其数值越大，表明吸附性能越好；*K*_F_、*n*为Freundlich吸附常数，*K*_F_值越大，表明吸附容量越高；1*/n*：异质性因子，当0<1/*n*<1时，其值越小，不均匀性越大，则印迹效果越强。

吸附动力学特性：取5 mg MIPs置于西林瓶中，加入2 mL CA标准溶液（10 ng/mL），室温下超声分散，放入摇床振荡，分别在不同时间点（2、4、6、8、10、30、60 min）取样，离心，吸取上清液，氮气吹干，残渣加流动相复溶，LC-MS测定CA含量，计算聚合物对CA的结合量，分别按准一级（式（4））和准二级动力学模型（式（5））拟合动力学吸附过程：


$$ln\;\left(q_{e}-q\right)=ln\;q_{e}-k_{1}t$$ (4)

可以变形为：


$$q=q_{e}\left(1-exp\left(-k_{1}t\right)\right)$$ (4-1)

$$\frac{t}{q}=\frac{1}{k_{2}{q_{e}}^{2}}+\frac{1}{q_{e}}t$$ (5)

其中，*q*_e_为吸附饱和时聚合物的吸附容量（mg/g），*t*为吸附时间（min），*q*为某时间点聚合物的吸附容量（mg/g），*k*_1_为准一级吸附平衡常数，*k*_2_为准二级吸附平衡常数。

吸附选择性：将MIPs加入到待吸附的COL标准溶液（60 μg/mL，5 mL）中，室温下振荡吸附2 h，离心，取上清液，HPLC法测定药物质量浓度。按式（6）~（8）分别计算分配系数（*K*_d_）、选择性系数（*α*）和相对选择系数（*β*）：


$$K_{d}=Q_{e}/C_{e}$$ (6)

$$a=K_{d\left(template\right)}/K_{d\left(interferent\right)}$$ (7)

$$\beta =\alpha_{\left(MIPs\right)}/\alpha_{\left(NIPs\right)}$$ (8)

其中，*K*_d（template）_为目标物的分配系数，*K*_d（interferent）_为干扰物的分配系数。

重复利用性：将MIPs加入到CA标准溶液（5 mL，50 μg/mL）中，室温静置吸附30 min，测定吸附后溶液中CA的质量浓度，计算吸附容量。将吸附饱和的MIPs转移至甲醇（含20%乙酸）中，室温静置解吸30 min；解吸后的MIPs再次加入CA标准溶液（5 mL，50 μg/mL），室温吸附30 min，LC-MS法测定CA的质量浓度并计算吸附容量。重复上述吸附-解吸步骤多次。

### 1.5 MIPs用于含药血浆的测定

取血浆样品100 μL，置于1.5 mL离心管中，加入-20 ℃预冷的甲醇300 μL、加入已知质量浓度的CDCA/CA标准溶液（5、50、500 ng/mL）100 μL，加入内标溶液（人参皂苷Rg1，100 ng/mL）10 μL，涡旋振荡3 min，离心15 min（12 000 r/min，4 ℃），吸取上清液于西林瓶中，35 ℃水浴下氮气吹至干。残渣加入MIPs的丙酮溶液，置于摇床摇匀30 min（300 r/min），过膜，吸取上清液，氮气吹至干，加流动相0.1%甲酸水-甲醇（30∶70，体积比）100 μL复溶，涡旋振荡1 min，离心15 min（15 000 r/min，4 ℃），取上清液，进样检测。

## 2 结果与讨论

### 2.1 MIPs材料表征

SEM观察结果显示，所制备的MIPs为粒径约1 μm的球形颗粒，粒径分布均匀（图2a）。TEM观察进一步发现，与NIP（图2b）相比，MIPs（图2c）的颗粒粒径明显减小，同时表面粗糙程度增加。

**图2:**
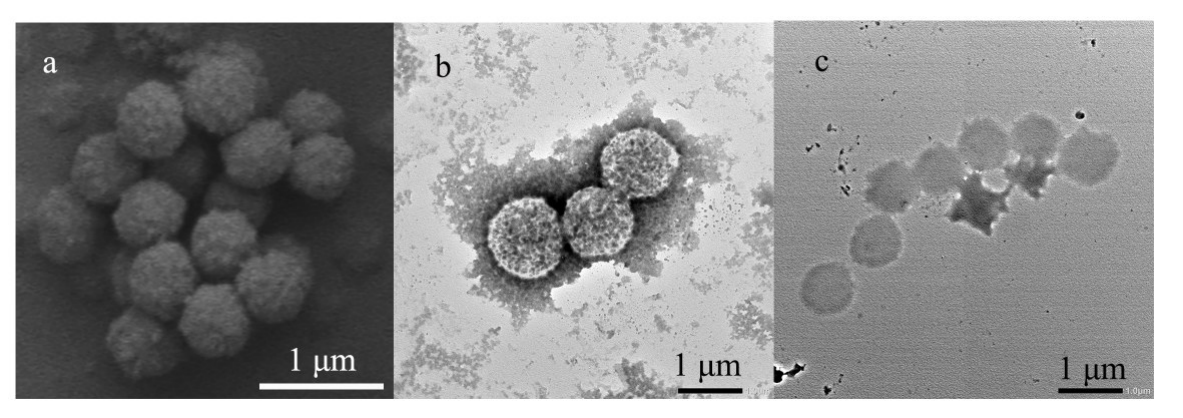
（a）MIPs的SEM图谱及（b）NIP和（c）MIPs的 TEM图谱

FT-IR分析结果（图3）显示，在MIPs的图谱中，可见羰基伸缩振动吸收峰较CA发生了红移，并出现1 144.3 cm^-1^和1 159.9 cm^-1^的C-O振动峰，表明MIPs中的模板分子CA被成功洗脱去除。与此同时，MAA和EGDMA的C=O伸缩振动分别由1 691.9 cm^-1^和1 723.1 cm^-1^位移至1 733 cm^-1^，而2 929.8 cm^-1^处MAA的COOH伸缩振动以及1 639.7 cm^-1^、1 637.1 cm^-1^处EGDMA和MAA的C=C伸缩振动峰均消失，表明MAA与EGDMA成功聚合，且在MIPs中存在CA和MAA分子间的氢键相互作用。

**图3:**
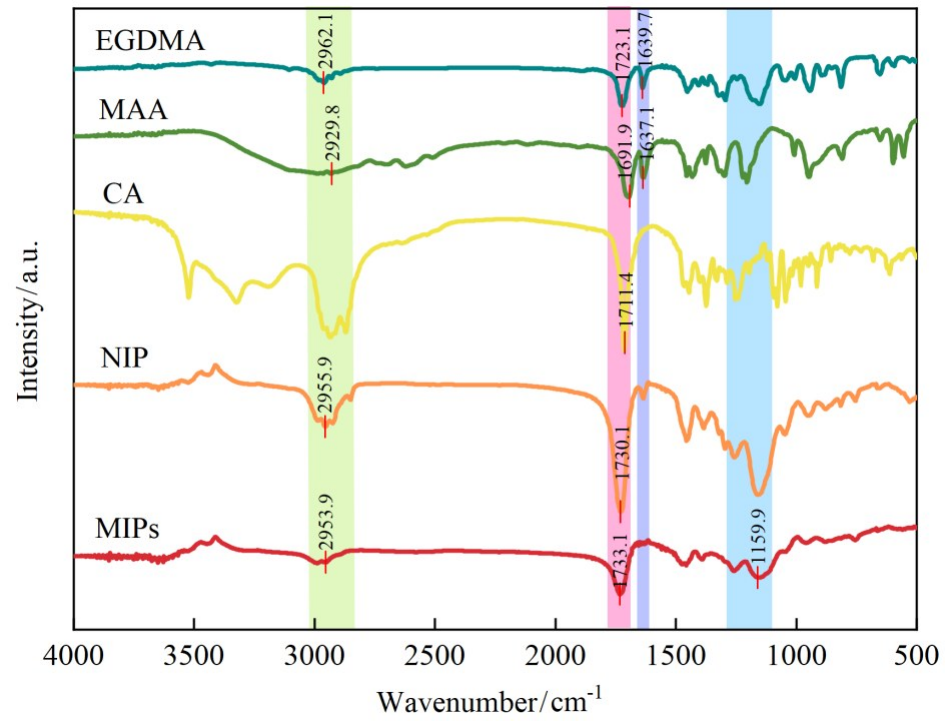
MIPs及相关原料的红外光谱图

### 2.2 MIPs吸附性能研究

为评估MIPs对CA的吸能性能，在室温条件下测定其等温吸附和动力学吸附能力；其中等温吸附实验数据采用Langmuir和Freundlich 等温吸附模型进行拟合分析，动力学吸附实验数据采用准一级动力学和准二级动力学模型拟合。如图4a所示，MIPs在相同条件下对CA的吸附量在0~100 ng/mL范围内随CA浓度增大而增大，最大吸附容量为0.16 μg/mg；而NIPs最大吸附容量为0.001 5 μg/mg，远低于MIPs。在Langmuir模型（ Y=18.59X/(1+0.072X) ，*R* ^2^=0.988 9）中，MIPs理论最大吸附容量为0.26 μg/mg；如图4b所示，吸附过程符合Freundlich模型（ $Y=29.71X^{0.5}$ ，*R* ^2^=0.998 8），为不均匀的化学吸附。如图4c所示，室温下MIPs对CA的吸附容量进行准一级（*Y*=18.17×（1-exp（-2.28*X*）），*R* ^2^=0.626 5）和准二级动力学（*Y=*0.001 6+0.055*X*，*R* ^2^=0.999 9）拟合，准二级动力学拟合结果*R* ^2^大大趋近于1，说明其吸附过程更符合准二级动力学模型。

**图4:**
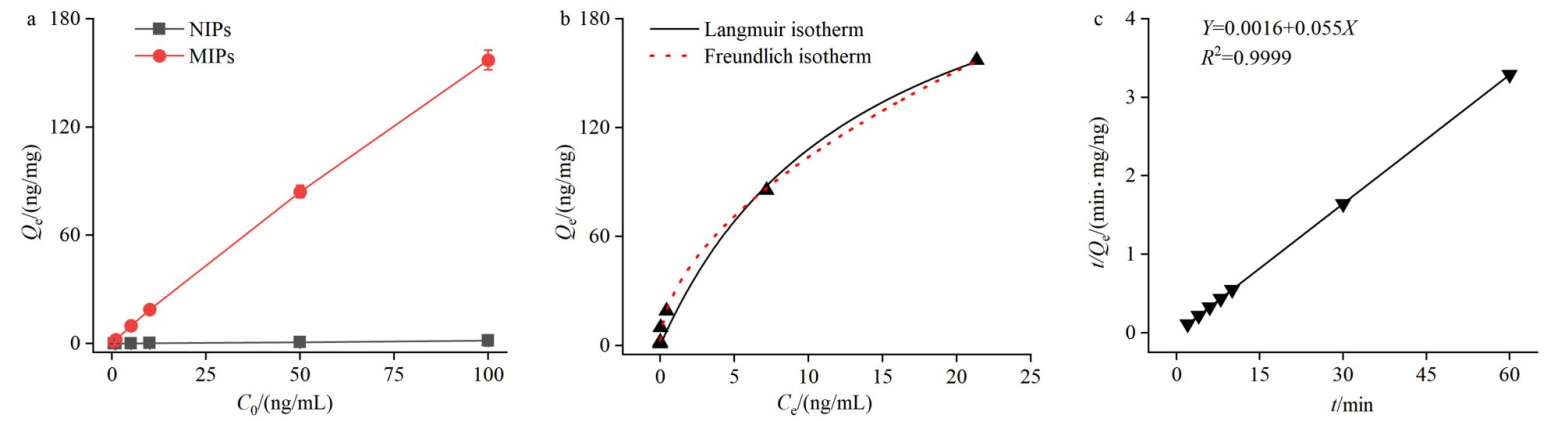
（a）平衡吸附等温线、MIPs的（b）等温吸附模型及（c）吸附动力学曲线

### 2.3 MIPs吸附选择性评价

实验选择在相同条件下针对不同化学结构的目标分子进行吸附实验，计算IF、*α*和*β*，评价MIPs的吸附选择性。如图5所示，目标分析物包括CA、CDCA及其结构类似物DXMS，以及具有显著结构差异的常见药物GA和COL。如表2所示，MIPs 对CA和CDCA的IF分别为4.99和4.88，显著高于其他分析物，提示MIPs上所形成的印迹位点与胆汁酸系列分子结构高度匹配，因此对胆汁酸系列化合物具有良好的特异性吸附能力。以吸附量最大的CA为对照计算的吸附选择性系数*α*随化合物结构差异显著增大至超过30，反映MIPs上的印迹位点确实能够特异性识别并吸附模板分子。在相同条件下，NIPs对于CA和其他化合物的吸附无显著差异，进一步表明MIPs的特异性识别能力源于其中存在的分子印迹位点。

**图5:**
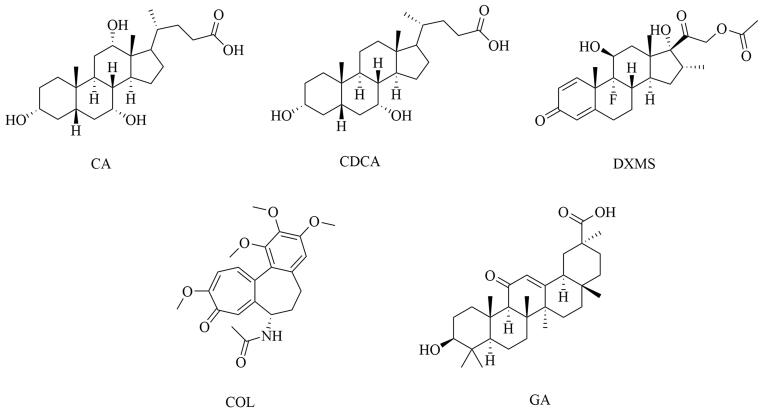
MIPs选择性实验的相关化合物结构式

**表2:** 吸附选择性参数

Analyte	MIPs			NIPs			IF	*β*
	*Q*_e_/（mg/g）	*K*_d_/（mg/mL）	*α*	*Q*_e_/（mg/g）	*K*_d_/（mg/mL）	*α*		
CA	14.32	1.00	-	2.87	0.067	-	4.99	-
CDCA	10.51	0.44	2.27	2.15	0.048	1.39	4.88	1.02
DXMS	4.50	0.11	8.68	1.65	0.036	1.86	2.73	1.82
GA	1.93	0.043	23.44	1.53	0.033	2.02	1.26	3.96
COL	1.51	0.033	30.54	1.59	0.034	1.94	0.95	5.25

IF=*Q*_e（MIPs）_/*Q*_e（NIPs）_.

### 2.4 MIPs应用效能评价

首先通过多次吸附-解吸附实验考察材料的重复使用性能。结果表明，经6次循环使用后，MIPs仍能保持84.6%的初始吸附容量，显示出良好的结构稳定性，可重复应用于复杂生物样本中目标分子的选择性分离富集。

为评估MIPs在含药血浆中的选择性吸附能力，采用同一批次MIPs对高中低不同质量浓度（5~500 ng/mL）的CA/CDCA含药血浆进行吸附实验。结果见表3，MIPs对CA和CDCA的吸附能力在加标量较低时，测得回收率较差，但在加标量为50~500 ng/mL时的绝对回收率为90%~100%，RSD<1.0 %，表明MIPs能够选择性识别并稳定富集目标分子。研究结果表明，所制备的MIPs对于胆酸、鹅去氧胆酸显示出优异的选择性富集能力，为血浆等复杂生物样品中微量胆汁酸类成分分析提供了一种便捷、高效的前处理方法。

**表3:** MIPs的应用效能评价（*n*=3）

Analyte	Content/（ng/mL）	Added/（ng/mL）	Found/（ng/mL）	Recovery/%	RSD/%
CA	598.2	5	601.4	64.0	0.4
	598.2	50	645.5	94.6	0.3
	598.2	500	1098.3	100.0	0.1
CDCA	90.3	5	94.0	74.0	2.6
	90.3	50	136.0	91.4	1.0
	90.3	500	589.9	99.9	0.6

## 3 教学实践与反思

### 3.1 教学组织实施

根据药学综合创新实验课程的总体教学目标，本实验建议安排16学时，采用线上线下混合教学模式，由教师引导有序组织开展课前（3学时）、课中（10学时）和课后学习（3学时），具体实施如下。

（1）课前。学生通过线上课程平台提前了解实验的具体内容和要求，预习MIT及其应用的相关文献，观看MIPs材料制备的演示视频，学习分析仪器操作，拟定实验方案，旨在培养学生的独立思考能力和创新思维。

（2）课中。采用任务驱动式教学方法。根据教学内容分成4个任务小组，在教师指导下分工协作，实时分享实验进展和发现。其中，①材料表征，利用SEM、TEM和FT-IR对MIPs进行测试，分析MIPs的形成机制；②吸附性能研究，利用LC-MS法测定材料的吸附等温线和吸附动力学，进行数据处理和吸附过程的模型拟合，分析MIPs的吸附机制；③吸附选择性评价，采用HPLC法测定材料对各种不同结构化合物的选择性系数和印迹因子等关键吸附选择性参数，定量评价MIPs的吸附选择效能；④应用效能评价，测定MIPs材料在经过反复循环使用后的饱和吸附量变化，并应用于模拟含药血浆样品的预处理，通过专属性和加标回收试验评价材料应用于实际样品中微量CA分离富集的效能。

（3）课后。教师组织各组整理、汇总实验结果，开展分组汇报、答辩和专题研讨；所有学生按要求完成实验报告。

### 3.2 教学反馈

实验结束后，通过问卷等方式及时了解学生对实验学习的评价和收获反馈，结果充分反映了学生对于药学综合创新实验的极大兴趣和积极反响。学生普遍认为，该实验融合了分子印迹聚合物材料的合成及其在复杂生物样品中微量组分分析研究中的应用，同时具有高阶性、创新性和趣味性。学生通过文献调研学习，了解到学科领域的前沿研究成果和发展趋势，显著拓宽了学术视野，激发了创新探索的意识和热情，并在自主研读文献、协作解决实验问题的过程中，促进学生构建“项目背景分析→文献资料调研→研究方案制订与组织实施→结果汇总与报告”的科研逻辑思维框架。

综上，本实验教学达成了预期目标，在增强学生药学专业知识和技能的同时，激发了科学研究热情，自主学习能力、沟通合作能力、综合分析能力和创新实践能力获得全面提升。

### 3.3 教学反思

（1）本实验是面向药学专业学生探索设计的一项科研综合创新实验。教学团队从当前的药学专业人才培养目标需求出发，结合针对新兴分子印迹聚合物的科研积累，凝练兼具高阶性、创新性、挑战度和可操作性的课题进行教学设计。基于上课学生的学情特点，本实验筛选学生熟悉的重要内源性代谢物胆酸作为分析对象，以解决在药物代谢组学研究中普遍存在的分析困难为目标，充分体现了药学科研综合创新实验的跨学科和科产教深度融合设计特点。

（2）本实验涉及分子印迹聚合物材料设计与合成制备、表征测试和应用效能评价等三个密切相关又相对独立的教学内容，相应采用线上线下混合的教学模式和任务驱动式的教学方法组织教学活动，按任务模块分组开展实验，同时利用线上课程平台进行辅助学习。具体来说，教师筛选分子印迹技术及其应用的相关教学资源发布到线上平台，供学生预习了解相关背景知识和实验具体内容与要求，理解本实验的两个关键知识点：1）MIT是通过模拟抗体-抗原特异性识别机制从而形成具有“分子记忆”效应的特异性识别位点的功能聚合物制备技术；2）MIPs能够在复杂基质中精准记忆目标分子的尺寸、形状和官能团分布的优异的目标分子识别性能，且耐受极端pH和温度条件。其中，针对耗时较长的MIPs材料制备，由教师提前安排完成并提供样品和演示视频，学生利用线上平台学习材料制备方法和有关分析仪器的操作使用，并制订后续分组实验方案。在线下教学活动组织过程中，教师主要发挥引导和反馈作用，帮助学生理解不同任务模块间的联系，以及如何应用知识解决实际问题，培养学生的创新思维和综合素质。

（3）实验结束后，组织学生从实验选题设计到组织实施和结果呈现进行全面、深入的讨论和反思，并提出改进建议，形成完整的闭环教学过程，通过对不同模块专业知识的系统整合，促进专业能力培养和科学思维形成。与此同时，紧扣国家战略和人类健康事业需求引导学生的主动反思，进一步将育人理念与创新驱动发展的国家战略紧密联系，促进思政元素的有机融合贯穿专业教育始终，强化对药学专业学生创新精神和科技强国的爱国情怀培养，实现“课程思政”育人的课程教学目标。

## 4 结论

针对药物分析领域复杂生物样品分析的前处理技术难题，我们选择以具有重要生理意义的内源性代谢物胆酸作为分析对象，设计了一项基于先进分子印迹技术应用的药学综合创新实验。本实验有机融合了药物分析和高分子化学、分析化学等多个药学相关领域的理论和技术，充分体现了课程资源的高阶性、创新性和挑战度。采用线上线下混合的教学模式和任务驱动式的教学方法，有效组织开展涵盖分子印迹聚合物材料合成、结构表征与应用效能评价全过程的实验教学活动，通过实践教学与产业前沿及课程思政的有机融合，切实提升药学专业学生的创新思维和综合实践能力，同时激发学生的科研热情和职业责任意识，是全人教育的有益实践。
